# Irrigation and Fertilization Scheduling for Peanut Cultivation under Mulched Drip Irrigation in a Desert–Oasis Area

**DOI:** 10.3390/plants13010144

**Published:** 2024-01-04

**Authors:** Jianshu Dong, Xiaojun Shen, Qiang Li, Zhu Xue, Xianfei Hou, Haocui Miao, Huifeng Ning

**Affiliations:** 1 College of Water Conservancy Engineering, Tianjin Agricultural University, Tianjin 300392, China; dongjianshu_1010@163.com (J.D.); xuezhu@tjau.edu.cn (Z.X.); 2 Institute of Western Agriculture, The Chinese Academy of Agricultural Sciences, Changji 831100, China; 3 Institute of Economic Crops, Xinjiang Academy of Agricultural Sciences, Urumqi 830091, China; lq19820302@126.com (Q.L.); hou544805196@163.com (X.H.); mc09876@163.com (H.M.); 4 Key Laboratory of Crop Water Use and Regulation, Ministry of Agriculture and Rural Affairs, Institute of Farmland Irrigation of Chinese Academy of Agricultural Sciences, Xinxiang 453002, China

**Keywords:** peanuts, water and nitrogen fertilizer, yield, quality, water- and nitrogen-use efficiencies

## Abstract

The aim of this study was to investigate the impact of water and nitrogen regulation on the characteristics of water and fertilizer demands and the yield, quality, and efficiencies of the water and nitrogen utilization of peanuts cultivated under mulched drip irrigation in a desert–oasis region. The experiment, conducted in Urumqi, Xinjiang, centered on elucidating the response mechanisms governing peanut growth, yield, quality, water consumption patterns, and fertilizer characteristics during the reproductive period under the influence of water and nitrogen regulation. In the field experiments, three irrigation levels were implemented, denoted as W_1_ (irrigation water quota of 22.5 mm), W_2_ (irrigation water quota of 30 mm), and W_3_ (irrigation water quota of 37.5 mm). Additionally, two nitrogen application levels, labeled N_1_ (nitrogen application rate of 77.5 kg·ha^−1^) and N_2_ (a nitrogen application rate of 110 kg·ha^−1^), were applied, resulting in seven treatments. A control treatment (CK), which involved no nitrogen application, was also included in the experimental design. The results indicate a direct correlation between the increment in the irrigation quota and increases in farmland water-related parameters, including water consumption, daily water consumption intensity, and water consumption percentage. The nitrogen harvest index (*NHI*) demonstrated a higher value in the absence of nitrogen application compared to the treatment with elevated nitrogen levels. The application of nitrogen resulted in an elevation in both nitrogen accumulation and nitrogen absorption efficiency within pods and plants. When subjected to identical nitrogen application conditions, irrigation proved to be advantageous in enhancing water-use efficiency (*WUE*), nitrogen partial factor productivity (*NPFP*), and the yield of peanut pods. The contribution rate of water to pod yield and *WUE* exceeded that of nitrogen, while the contribution rate of nitrogen to nitrogen-use efficiency (*NUE*) was higher. The total water consumption for achieving a high yield and enhanced water- and nitrogen-use efficiencies in peanuts cultivated under drip irrigation with film mulching was approximately 402.57 mm. Taking into account yield, quality, and water- and nitrogen-used efficiencies, the use of an irrigation quota of 37.5 mm, an irrigation cycle of 10–15 days, and a nitrogen application rate of 110 kg·ha^−1^ can be regarded as an appropriate water and nitrogen management approach for peanut cultivation under mulched drip irrigation in Xinjiang.

## 1. Introduction

Peanut (*Arachis hypogaea* L.) holds significant economic importance as a cash crop in China, playing a pivotal role in the national economy. The development of peanut production plays an important role in alleviating the shortage of edible oil [1,2]. Xinjiang’s temperate continental, arid climate creates a distinctive natural ecological environment conducive to cultivating peanuts with high quality and high yield [3]. Water and fertilizer are two indispensable factors crucial for crop growth [4]. On one hand, irrigation significantly influences crop nitrogen uptake, translocation, and utilization [5]. On the other hand, the judicious application of supplemental nitrogen fertilizers can mitigate certain adverse effects on growth resulting from water deficit conditions [6]. The integrated management of water and nitrogen by capitalizing on the synergistic interaction of water and nitrogen can maximize yield and minimize the use of water resources, which is particularly important in Xinjiang [7,8]. With continuous and swift economic and social development, the issue at hand has emerged as a crucial factor constraining the renewed expansion of peanut production in Xinjiang. Therefore, how to explore reasonable water and nitrogen control indicators in Xinjiang is of great significance to promoting the sustainable development of local agriculture.

In the arid inland area of Northwest China, the synergistic interaction between water and fertilizer is an important means of ensuring food security and the sustainability of water resources. In recent years, many scholars have conducted substantial research on the efficient utilization of water and nitrogen in drip irrigation under mulch. Lv et al. [9] showed that under the condition of a continuous water shortage, the effect of nitrogen fertilizer was limited, and nitrogen accumulation in crops was significantly reduced. Increasing the application of nitrogen fertilizer would aggravate the reduction in the nitrogen-use efficiency and yield of crops. Li et al. [10] found that an increase in irrigation amount promoted the efficiency of nitrogen fertilizer, and a moderate increase in nitrogen fertilizer was beneficial to promoting an increase in yield through a coupling experiment involving water and nitrogen. Hu et al. [11] showed that nitrogen application had a significant effect on the water consumption of crops. Under the same irrigation method, increasing the amount of nitrogen fertilizer would significantly increase the water consumption of peanuts during the whole growth period. Xia et al. [12] observed that coupling water and fertilizer not only conserved water and enhanced yield but also improved the quality of peanuts. Therefore, the key to improving the water- and nitrogen-use efficiencies of crops in arid and semi-arid areas is to leverage the interaction effect of water and nitrogen, regulating the water-use process of crops through the reasonable, comprehensive management of water and nitrogen and promoting the effect of fertilizer on water while facilitating the regulation of water by fertilizer [13].

Previous studies mainly focused on the macroscopic effects of crop growth and yield and the physiological mechanism underlying these effects through qualitative analyses. However, due to the use of different experimental materials and control methods, the conclusions are inconsistent. Peanut cultivation in Xinjiang is in the initial stage of development; the soil in the area is barren, and there is a large demand for water and nitrogen fertilizer in actual production. There are few studies on the growth and yield of peanuts under the condition of drip irrigation with film mulching and the absorption and utilization of water and nitrogen in Xinjiang. Due to the lack of comprehensive consideration of yield along with water and nitrogen absorption and utilization, it is difficult to quantitatively determine a more effective nitrogen irrigation system to achieve the goal of water and fertilizer savings, high yield, and high quality. In this study, a field experiment was conducted to study the effects of different water and nitrogen conditions on water consumption characteristics and the nitrogen utilization of peanuts under mulched drip irrigation in Xinjiang and to clarify the regulatory effects of different water and nitrogen treatments on the water and nitrogen absorption and utilization of peanuts. The objective was to provide a theoretical basis for the efficient utilization of water and fertilizer for cultivating peanuts under mulched drip irrigation in Xinjiang and to promote the high-quality, efficient, green, and sustainable development of the peanut industry in Xinjiang.

## 2. Results

### 2.1. Effects of Water and Nitrogen Regulation on Water Consumption Characteristics of Peanuts under Mulched Drip Irrigation

To determine the suitable water and nitrogen indexes of peanuts under drip irrigation in Xinjiang, the water consumption, daily water consumption intensity, and water consumption modulus of peanuts under different water and nitrogen treatments were analyzed. It can be seen from Table 1 that the total water consumption and irrigation consumption of peanuts increased with an increase in irrigation, and the soil water consumption decreased with an increase in irrigation and nitrogen application. Under the W_1_ (irrigation water quota of 22.5 mm), W_2_ (irrigation water quota of 30 mm), and W_3_ (irrigation water quota of 37.5 mm) treatments, an increase in the nitrogen application rate resulted in a decrease in both irrigation consumption and total water consumption. Simultaneously, there was an increase in the proportion of irrigation consumption to total water consumption, coupled with a decline in the proportion of soil water storage consumption to total water consumption. This observation indicates that irrigation emerges as the primary source of water consumption for crops in the arid regions of Xinjiang.

Across the three nitrogen treatments, an elevation in irrigation volume resulted in a notable increase in the proportion of irrigation consumption to total water consumption, accompanied by a significant decrease in the proportion of soil water consumption to total water consumption. This observation underscores that moderate nitrogen application enhances peanuts’ capacity to utilize soil water storage under mulched drip irrigation. However, under certain nitrogen application conditions, an increase in irrigation volume is not conducive to peanuts’ efficient soil water utilization. The regression analysis revealed a strong and positive linear relationship between water consumption and irrigation volume, as evidenced by a coefficient of determination (*R*^2^) of 0.947 **.

Among the three water and nitrogen treatments, as the nitrogen application rate increased, the water consumption, water consumption intensity, and water consumption percentage of peanuts under drip irrigation with film mulching displayed a marginal downward trend after the flowering–pegging stage (Table 2). Notably, during the flowering–pegging stage, the water consumption intensity of CK (irrigation water quota of 30 mm, without the application of nitrogen), N_1_ (nitrogen application rate of 77.5 kg·ha^−1^), and N_2_ (nitrogen application rate of 110 kg·ha^−1^) increased by 139.63%, from 123.20% to 152.55%, and from 104.92% to 133.15%, respectively, compared to the water consumption intensity at the seedling stage. The reason might be that plants in the seedling stage were young and grew slowly, the temperature and photosynthetically active radiation were low, and the plant water consumption and water consumption intensity were at low levels. In the flowering–pegging stage, plant growth gradually flourished, the leaf area index of the plants increased rapidly, and the plants began to flower and pollinate. At the same time, their needles also needed to consume some water. The water consumption intensity increased by 0.69%, 0.17%~10.21%, and 0.78%~13.71% in the pod-setting stage compared to the flowering–pegging stage. Compared to the pod-setting stage, it decreased by 76.64%, 74.94%~77.00%, and 74.69%~77.15% at the pod-filling stage. In the pod-setting stage, the temperature and solar photosynthetic effective radiation were at high levels, and the soil evaporation and plant transpiration were intensified. Therefore, the water consumption and water consumption intensity of peanuts were high at this stage. Temperature and photosynthetically active radiation decreased during the pod-filling stage. At this stage, the morphological indexes of the plants were in a state of gradual decline, and the water consumption intensity decreased. Therefore, the proportion of water consumption during the whole growth period was low. The results indicate that an increase in the nitrogen application rate leads to modest increases in water consumption and water consumption intensity during the flowering–pegging and pod-setting stages. The water consumption percentage of peanuts ranged from 11.88% to 15.02% at the seedling stage, from 30.20% to 32.99% at the flowering–pegging stage, from 40.99% to 43.81% at the pod-setting stage, and from 11.69% to 13.49% at the pod-filling stage. The findings reveal that irrigation during the pod-setting stage significantly elevates the water consumption percentage coefficient of peanuts, thereby fostering an increase in water consumption during this stage. Similarly, the flowering–pegging stage contributes to an increased water consumption coefficient, enhancing the overall utilization of water by peanuts.

### 2.2. Effects of Water and Nitrogen Regulation on Yield and Water Use of Peanuts under Mulched Drip Irrigation

**Figure 1:**
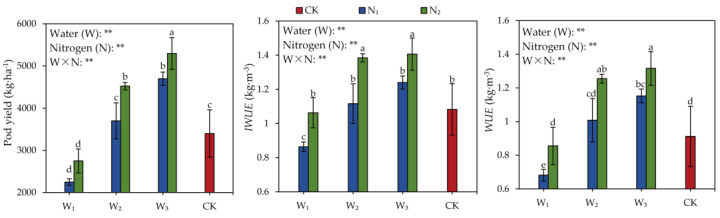
Effects of water and nitrogen regulation on peanut yield and water-use efficiency under mulched drip irrigation. Note: W_1_, W_2_, and W_3_ represent irrigation water quotas of 22.5, 30, and 37.5 mm, respectively. N_1_ and N_2_ represent nitrogen application rates of 77.5 and 110 kg·ha^−1^, respectively. CK represents a 30 mm irrigation water quota and no nitrogen application. For each index, the mean values within a column followed by a different letter are significantly different at *p* ≤ 0.05 according to the LSD test ** indicates significance at the 0.01 probability level, respectively; ns: non-significant.

Figure 1 illustrates that under the W_1_, W_2_, and W_3_ treatments, pod yield exhibited an ascending trend corresponding to the nitrogen application rate. In the W_2_ treatment, the yield with nitrogen application was 8.82~33.09% higher than without, suggesting that nitrogen application could enhance pod yield under specific irrigation conditions. It shows that ensuring irrigation is fundamental, and increasing the amount of nitrogen fertilizer to exert the coupling regulation effect of water and nitrogen on yield is the key to achieving a high yield of peanuts under drip irrigation in the arid areas of Xinjiang. For both the W_1_ and W_3_ treatments, the increase in pod yield with increasing nitrogen application rates was less pronounced, indicating that the impact of irrigation on yield improvement surpassed that of nitrogen. Under N_1_, the yields of W_2_ and W_3_ increased by 52.13~64.44% compared to W_1_. Similarly, under N_2_, the yields of W_2_ and W_3_ increased by 64.55~92.73% compared to W_1_, signifying that irrigation could boost yield with or without a certain level of nitrogen application. The reason might be that full irrigation promoted the net photosynthetic rate of peanut leaves, increased the output of photosynthetic products, and increased the physiological activities of various organs, thereby promoting the absorption and accumulation of nutrients by plants and ultimately affecting pod yield.

The modulation of water and nitrogen exhibits discernible differences in effects on peanut plants’ water-use efficiency (*WUE*) under mulched drip irrigation. Across the three irrigation treatments, noticeable upward trends in *WUE* and irrigation water-use efficiency (*IWUE*) accompanied the escalating nitrogen application rates, with a significant distinction between nitrogen and non-nitrogen applications. This observation suggests that under specific irrigation conditions, nitrogen application positively influences the enhancement in both *WUE* and *IWUE*. In the context of N_2_ treatment, the *WUE* and *IWUE* of W_3_ showed augmentations of 53.83% and 32.31%, respectively, compared to W_1_. This increase was not markedly different from that observed in W_2_. Under N_1_, no statistically significant distinctions were observed in *WUE* and *IWUE* among the three irrigation treatments. This underscores that augmenting the nitrogen application rate has the potential to boost pod yield under specific irrigation conditions. The N_2_ treatment in this experiment demonstrates the efficacy in enhancing yield through strategic fertilizer adjustment in conjunction with water application.

### 2.3. Effects of Water and Nitrogen Regulation on Nitrogen Utilization of Peanuts under Mulched Drip Irrigation

Significant interaction effects between water and nitrogen regulation were observed for pod nitrogen accumulation (*F* = 4.108, *p* ≤ 0.05), plant nitrogen accumulation (*F* = 31.676, *p* ≤ 0.01), nitrogen uptake efficiency (*UPE*) (*F* = 21.410, *p* ≤ 0.01), and nitrogen harvest index (*NHI*) (*F* = 15.619, *p* ≤ 0.01).

Trends in pod nitrogen accumulation, plant nitrogen accumulation, *UPE*, *NHI*, nitrogen-use efficiency (*NUE*), and nitrogen partial factor productivity (*NPFP*) varied under different water and nitrogen treatments, as indicated in Table 3. Under W_2_, pod nitrogen accumulation and plant nitrogen accumulation exhibited significant increases under the N_1_ and N_2_ treatments compared to the CK treatment, with increments ranging from 33.20% to 85.95% and from 21.38% to 186.62%, respectively. The *NHI* of CK surpassed that of N_2_ by 56.3% and was 8.10% lower than that of N_1_, with the highest *NUE*. Noteworthy differences were observed in the *UPE* and *NPFP* between N_1_ and N_2_. Specifically, the *UPE* of N_2_ was 66.67% higher than that of N_1_, while the *NPFP* was 13.83% lower. Under W_1_, the *UPE* of N_2_ exceeded that of N_1_ by 39.19%. The *NHI* of N_2_ showed a 0.56%~10.83% decrease compared to CK and N_1_, with no significant difference between the latter two treatments. For W_3_, pod nitrogen accumulation, plant nitrogen accumulation, *UPE*, and *NHI* under the N_2_ treatment surpassed those under the N_1_ treatment by 85.13%, 48.16%, 4.13%, and 24.47%, respectively. The *NUE* and *NPFP* under the N_2_ treatment were 23.91% and 20.54% lower than those under the N_1_ treatment. The reason might be that when too little nitrogen fertilizer was applied to the soil, the amount of nitrogen absorbed by the crop was less than the amount that entered the deep soil and accumulated, resulting in an increase in the leaching loss of nitrogen fertilizer. At the same time, excessive nitrogen application resulted in more nitrogen and photosynthetic products being accumulated in the stems and leaves of vegetative organs in the late growth stage, which was not conducive to the transport of photosynthetic products to grains in the late growth stage.

Under N_1_, an escalation in irrigation amount led to elevated pod nitrogen accumulation, plant nitrogen accumulation, and *UPE* for W_2_ and W_3_ compared to the W_1_ treatment, with increases ranging from 96.55% to 118.18%, from 64.56% to 79.62%, and from 63.51% to 78.38%, respectively. No significant differences were observed in terms of *NHI*. Notably, the *NUE* and *NPFP* of W_3_ surpassed those of W_1_ and W_2_, exhibiting increments of 26.95~38.66% and 27.01~108.86%, respectively, under N_1_. Under N_2_, except for plant nitrogen accumulation, other nitrogen-related indices exhibited the highest values under W_3_, with the plant nitrogen accumulation of W_2_ being 73.97~112.48% higher than W_1_ and W_3_. Therefore, under irrigation conditions, nitrogen application contributes to nitrogen accumulation in pods and plants, enhancing *UPE*. Moderate irrigation levels promote an increase in *NHI*. However, excessive nitrogen application can lead to a reduction in both *NUE* and *NPFP*. The reason may be that sufficient water promotes nitrogen absorption and transport, promotes the re-transport of carbohydrates stored in vegetative organs to grains, and significantly increases *NUE*. Increasing the application of nitrogen fertilizer by a certain amount can promote the utilization of soil moisture and improve the *UPE* of peanuts. However, insufficient water limits the normal function of nitrogen fertilizer, and excessive nitrogen application has little compensation effect on irrigation. In our experiments, the W_3_N_2_ (irrigation water quota of 37.5 mm; nitrogen application rate of 110 kg·ha^−1^) treatment resulted in higher nitrogen accumulation levels and a superior *NHI*.

### 2.4. Regression Analysis of Peanut Yield, Water Consumption, and Water- and Nitrogen-Use Efficiencies under Mulched Drip Irrigation

The differences in water and nitrogen uptake by peanuts under drip irrigation result in varying impacts on total water consumption, *WUE*, *NUE*, and pod yield. As revealed by the multiple linear regression analysis (Table 4), water-related factors contribute significantly to total water consumption (98.5%), *WUE* (83.7%), *NUE* (45.6%), and pod yield (92.7%). Conversely, nitrogen-related factors contribute to a lesser extent, with rates of 16.9%, 34.4%, 50.5%, and 22.6%, respectively. The primary determinants of total water consumption, *WUE*, and pod yield for peanuts under drip irrigation with film mulching are water-related factors. In contrast, nitrogen-related factors play a more prominent role in determining the *NUE*.

The correlation analysis among the indices reveals that an increase in total water consumption corresponds to an upward trend in both *WUE* and *NUE* within a specific range of water consumption. Total water consumption exhibits a significant positive correlation with pod yield, with determination coefficients of 0.976 and 0.895, respectively. *WUE* also displays a significant positive correlation, with a determination coefficient of 0.767. However, there is no significant positive correlation between *NUE* and total water consumption, and the coefficient of determination is 0.442. This suggests that multiple factors influence *WUE* and *NUE*. The coupling of water and nitrogen can enhance peanut pod yield within defined ranges of irrigation and fertilization.

### 2.5. Effects of Water and Nitrogen Regulation on Peanut Quality under Mulched Drip Irrigation

Protein and oil contents serve as crucial benchmarks for evaluating the quality of peanuts, with the protein content influencing the extraction yield of peanut protein and the oil content determining oil production. The impact of diverse irrigation and nitrogen treatments on the quality of peanut kernels is outlined in Table 5.

Table 5 illustrates that water treatment significantly influences protein and oil contents, whereas nitrogen treatment exhibits no noteworthy effect on peanut quality. At the same nitrogen level, the protein contents of the W_1_ and W_2_ treatments significantly surpassed that of the W_3_ treatment. However, at the same irrigation level, there was no substantial difference in protein content among the CK, N_1_, and N_2_ treatments. The influence of irrigation and nitrogen application on oil content contrasted with that of protein content. Specifically, the oil content of the W_3_ treatment was significantly higher than the oil contents of W_1_ and W_2_, while the oil content of the N_1_ treatment was lower than that of the N_2_ treatment. These results imply that an elevated water content diminishes the protein content of peanut kernels while concurrently increasing the oil content of the kernels. The reason may be that an increase in water has a diluting effect on the protein content of peanut kernels.

### 2.6. Comprehensive Evaluation Model for Peanut Growth under Mulched Drip Irrigation

**Figure 2:**
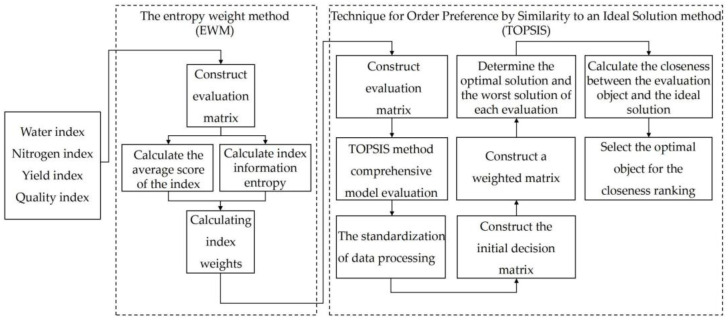
Structure block diagram of a comprehensive evaluation model for peanut growth under mulched drip irrigation.

The effects of the synergistic regulation of drip irrigation and fertilization on peanut yield, yield composition, water- and nitrogen-use efficiencies, and kernel quality were analyzed in our experiments. The entropy weight method was used to obtain the weight of each single index. A comprehensive evaluation model for peanut growth with high efficiency, high yield, and high quality was established by using the technique for order preference by similarity to an ideal solution (TOPSIS) (Figure 2) to find the best water and nitrogen regulation system for peanut planting. The comprehensive evaluation indexes were *WUE* (x_1_), *IWUE* (x_2_), *NUE* (x_3_), *NHI* (x_4_), pod yield (x_5_), 100-pod weight (x_6_), 500 g pod number (x_7_), kernel rate (x_8_), 100-kernel weight (x_9_), pod per plant (x_10_), pod weight per plant (x_11_), protein (x_12_), and oil content (x_13_).

The entropy weight method is a commonly used weighting method. The basic idea is to determine the weight according to the attributes and characteristics of the evaluation index itself. This method is not affected by human subjective factors and can scientifically and reasonably determine the weight based on the evaluation object’s attributes. The weight of each evaluation index of peanuts calculated using the entropy weight method and are shown in Table 6. The weights of the peanut indicators in descending order were the 100-pod weight (x_6_), 100-kernel weight (x_9_), protein (x_12_), pod weight per plant (x_11_), kernel rate (x_8_), pod yield (x_5_), oil content (x_13_), *WUE* (x_1_), 500 g pod number (x_7_), *NUE* (x_3_), pod per plant (x_10_), *IWUE* (x_2_), and *NHI* (x_4_).

TOPSIS is a common method of solving the problem of multi-objective decision analysis. It finds the optimal target and the worst target (expressed as the ideal solution and negative ideal solution, respectively) in multiple targets and sorts them according to the closeness degree of the ideal solution. The closeness degree is between 0 and 1, and the closer the value is to 1, the closer the corresponding evaluation target is to the optimal level. On the contrary, the closer the value is to 0, the closer the evaluation target is to the worst level. Based on the TOPSIS comprehensive model, a comprehensive evaluation of each index was carried out. After the evaluation indices were combined and weighted, a weighted normalized evaluation matrix based on the combined weights was established, and the ideal solution and closeness degree of each index were calculated. According to TOPSIS, the results of each treatment are shown in Table 7. The comprehensive index of the W_3_N_2_ treatment was the largest, which was 0.7424. Under this treatment condition, the comprehensive evaluation of peanuts was the best, followed by the W_3_N_1_ (irrigation water quota of 37.5 mm; nitrogen application rate of 77.5 kg·ha^−1^) treatment, while the W_1_N_1_ (irrigation water quota of 22.5 mm; nitrogen application rate of 77.5 kg·ha^−1^) treatment was the worst, with a comprehensive index of only 0.2112.

## 3. Discussion

### 3.1. Effects of Water and Nitrogen Regulation on Water Consumption Characteristics of Peanuts under Mulched Drip Irrigation

The change rule of crop water consumption has a certain relationship with soil environment [14,15,16], climatic conditions [17,18], cultivation system [19,20], etc. The water consumption of each stage of crop growth and development can directly represent the water consumption characteristics and water demand of crops and can reflect the sensitivity of crops to water in each growth period, which can be used to infer the critical period and peak period of crop water demand to promote the growth and development of crops to increase yield [21]. This experimental study shows that with an increase in irrigation amount, the proportion of irrigation consumption to total water consumption increases, and the proportion of soil water consumption to total water consumption decreases, which is similar to the results reported by Liu et al. [22]. In this study, the water consumption of peanuts increased with an increase in irrigation amount and decreased slightly with an increase in nitrogen application amount, and the total water consumption of peanuts in each treatment was greater than the consumption of the irrigation amount, indicating that irrigation was the main reason for the difference in water consumption. The variation in water consumption intensity at different growth stages was as follows in descending order: pod-setting stage (approximately 4.19 mm·d^−1^), flowering–pegging stage (approximately 4.01 mm·d^−1^), seedling stage (approximately 1.76 mm·d^−1^), and pod-filling stage (approximately 1.01 mm·d^−1^). The water consumption modulus during the seedling and full fruit stages decreased with a decrease in irrigation amount, but it did not show a regular change trend compared with water consumption and daily water consumption intensity. This is because the water consumption modulus is determined by many factors, such as environmental conditions, water consumption during the whole growth period and stage, and the duration of growth period [23]. At the seedling stage, the temperature is low, plants grow slowly and are short, their leaves are smaller, plant transpiration is small, and the water consumption modulus is small. At the flowering–pegging stage, the temperature, photosynthetically active radiation, and sunshine hours reach their peak, the plant growth process accelerates, the growth of peanuts begins to transition from vegetative growth to reproductive growth, the transpiration of leaves and plants increases rapidly, and the water consumption and water consumption modulus increase significantly. The pod-setting stage of reproductive growth and vegetative growth is a critical period of water demand in peanuts. At this stage, temperature and photosynthetically active radiation are still at high levels, peanut plants grow and develop robustly, and the water consumption and water consumption modulus each reach a maximum. Then, at the pod-filling stage, due to the gradual maturity of peanuts, the gradual cessation of vegetative growth, and the decrease in temperature, the water consumption of peanuts gradually decreases, and the water consumption modulus also decreases. This research conclusion aligns with the findings of Shen et al.’s study on cotton [24]. In this study, the water consumption characteristics of peanuts at different growth stages are fully considered, a reasonable water deficit condition is evaluated during the seedling stage and the full-fruit stage; and excess water after the normal growth of peanuts is transferred to the peak periods of water demand, such as during the pod-setting stage and flowering–pegging stage, to achieve the purpose of saving water and increasing production.

### 3.2. Effects of Water and Nitrogen Regulation on Yield and Water-Use Efficiency of Peanuts under Mulched Drip Irrigation

The key to water and nitrogen regulation is to promote nitrogen utilization via water regulation and to regulate water via nitrogen application, as well as to improve crop yield and water- and nitrogen-use efficiencies through the interaction between water and nitrogen [25]. Yield and water-use efficiency are the primary indicators that determine the economic benefits of peanut cultivation [26]. This study showed that the interaction of water and nitrogen had a significant effect on peanut yield. The yield, water-use efficiency, and irrigation water-use efficiency decreased with a decrease in the fertilizer application rate, indicating that an increase in the fertilizer application rate to a certain range was beneficial for promoting increases in yield and the absorption and utilization of water by plants; these results are consistent with the results reported by Li [27] and Wu et al. [28]. The reason may be that reasonable water and fertilizer application can reduce the ineffective evaporation of field plants and improve water-use efficiency [29]. The findings indicate that optimal conditions were achieved with an irrigation quota of 37.5 mm and a fertilizer application rate of 110 kg·ha^−1^. These conditions resulted in the highest peanut yield, water-use efficiency, and irrigation water-use efficiency, measured at 5297.35 kg·ha^−1^, 1.32 kg·m^−3^, and 1.41 kg·m^−3^, respectively, thus accomplishing the goal of a stable yield and high efficiency. Moreover, it is noteworthy that the irrigation amount in this experiment was relatively low, and the optimal nitrogen application level was lower than that used in other regions. In subsequent experiments, there is a potential to explore the interaction effects of water and nitrogen under higher irrigation conditions by incrementally increasing the irrigation volume. This investigation involving diverse water and nitrogen ratios under drip irrigation with film mulching is ongoing. This research aims to identify precise water and nitrogen indicators to contribute to Xinjiang’s continuous effort to enhance peanut yield.

### 3.3. Effects of Water and Nitrogen Regulation on Nitrogen Utilization of Peanuts under Mulched Drip Irrigation

Irrigation amount and the irrigation period have significant regulatory effects on the nitrogen absorption and utilization of peanut plants [30]. With increasing irrigation levels, there are observed increases in nitrogen accumulation in pods, plant nitrogen accumulation, nitrogen uptake efficiency, nitrogen harvest index, nitrogen-use efficiency, and nitrogen partial factor productivity. As nitrogen application rates increase, there are decreases in the nitrogen harvest index, nitrogen-use efficiency, and nitrogen partial factor productivity. These findings are consistent with the research conclusions of Wang et al. [31] on wheat and Jiang et al. [32] on rice. The observed increases in the nitrogen harvest index and nitrogen utilization rate under the CK treatment compared to N_1_ and N_2_ can be primarily attributed to the peanut crops’ utilization of nitrogen in the soil. Elevated water stress weakens the impact of nitrogen fertilizer on peanut nitrogen uptake, while increased nitrogen fertilizer, in turn, diminishes the efficiency of peanut nitrogen utilization [33]. Compared to W_2_, although W_1_ promoted the efficient utilization of nitrogen by peanuts, it was not conducive to the efficient utilization of resources due to the subsequent low economic yield. Under W1, by increasing the supply of nitrogen fertilizer, the nitrogen-use efficiency decreased, and its translocation amount, translocation efficiency, and proportion in nitrogen fertilizer production efficiency decreased. Under W2 with nitrogen application, the yield was 8.82%~33.09% higher than that of W2 with no nitrogen application, indicating that appropriate nitrogen application promoted the distribution of more nitrogen to reproductive organs and a better regulation of population quality, which was conducive to promoting peanut yield and improving nitrogen-use efficiency. Under W3, high nitrogen led to a decrease in nitrogen-use efficiency, and the nitrogen-use efficiency of the treatment with a low nitrogen level was the highest [34]. Reasonable water and nitrogen coupling promoted the transfer of nutrients to increase economic yield and nitrogen-use efficiency. This study showed a significant increase in nitrogen accumulation in both peanut plants and pods with an increase in the nitrogen application rate. Some studies have suggested that when the nitrogen application rate is 225 kg·ha^−1^, the nitrogen-use efficiency decreases with an increase in the nitrogen application rate [35]. The optimal nitrogen application rate was 110 kg·ha^−1^ in this experiment, and this might be due to different crops. The inconsistent demand for nitrogen fertilizer might also be due to the fact that the nitrogen fertilizer treatment was smaller in amount in this experiment. Nitrogen fertilizer gradient treatments can be further studied in future research.

### 3.4. Regression Analysis of Peanut Yield, Water Consumption, and Water- and Nitrogen-Use Efficiencies under Mulched Drip Irrigation

In agricultural production, water- and nitrogen-use efficiencies are influenced by many factors [36,37]. All variables capable of impacting peanut pod yield, water consumption, and nitrogen levels will inevitably exert a direct or indirect influence on the overall efficiencies of water and nitrogen utilization. In our experiment, under the W_2_ condition, the application of nitrogen led to a notable increase in yield, ranging from 8.82% to 33.09% when compared to treatments with no nitrogen application. Nitrogen application also facilitated an enhancement in water-use efficiency. Under uniform nitrogen application rates, irrigation emerged as a beneficial factor in augmenting peanut pod yield and improving nitrogen fertilizer production efficiency. Within specified ranges of irrigation and nitrogen application, the proportional contribution of water to pod yield and water-use efficiency surpassed that of nitrogen. The contribution of nitrogen to nitrogen-use efficiency exhibited a higher ratio. The quantities of water and nitrogen fertilizer demonstrated a consistent threshold range. A synergistic relationship between water and nitrogen was observed within this range, signifying a coupling effect. Maintaining an appropriate nitrogen application rate through adaptive management proved instrumental in enhancing the peanut plants’ capacity to utilize soil water storage effectively. This adaptive approach diminished dependence on irrigation, compensating for insufficient irrigation’s adverse impact on peanut pod yield. These findings underscore the importance of strategic nitrogen management in optimizing water- and nitrogen-use efficiencies in peanut cultivation.

### 3.5. Effects of Water and Nitrogen Regulation on Peanut Quality under Mulched Drip Irrigation

The quality of peanuts determines the edible value and economic benefits of kernels. Therefore, attention should be paid to the quality of kernels in addition to yield [38]. Reasonable irrigation treatment can improve crop quality. A moderate, regulated water deficit is beneficial to the formation of protein and the accumulation of fat in crops [39]. Studies have shown that an increase in water has a diluting effect on the protein content of seed kernels [40]. This is consistent with the results of this study; at each irrigation level, the protein content of peanuts grown under the high-water treatment was lower than that under middle- and low-water treatments, while the oil content was higher than that under middle- and low-water treatments. The reason is that too much irrigation is not conducive to the dissolution of nitrogen fertilizer in the soil into nitrogen that can be absorbed and utilized by crops, thus inhibiting the synthesis of amino acids in kernels. Amino acids are the basic units of protein which convert plastic substances in peanut kernels into protein. Luan et al. [41] reported a negative correlation between the oil and protein contents in peanut kernels, aligning with our quality correlation analysis outcomes. Simultaneously, the application of nitrogen fertilizer in our study resulted in an augmentation in oil content in peanut kernels, thereby fostering the enhancement of economic returns associated with peanuts and facilitating their storage and processing. The elevation in oil content corresponded with increased irrigation levels, which could possibly be attributed to the promotion of nitrogen transport with higher irrigation amounts, consequently stimulating the synthesis of oil content.

### 3.6. Comprehensive Evaluation Model for Peanut Growth under Mulched Drip Irrigation

A high yield is the goal of farmers, and high quality is demanded by consumers. *WUE* and *NUE* are the core components of the efficient use of agricultural water and fertilizer resources. However, in actual production, it is difficult to achieve maximum output, quality, *WUE*, and *NUE* at the same time. In this study, the TOPSIS method was used to evaluate the yield, *WUE*, *NUE*, and quality of peanuts cultivated under seven different treatments. This method can provide effective solutions for the optimization of different traits through a comprehensive evaluation of the target populations. This study improved the traditional TOPSIS method and adopted the entropy method to determine the weight of each evaluation index, thus enhancing the reliability and rationality of the evaluation results. The overall benefit of the W_3_N_2_ treatment was the largest. Therefore, the coupling of water and nitrogen for cultivating peanuts not only resulted in increased yields, *WUE*, and *NUE* but also improved nutritional quality. The results of this study provide a practical reference for peanut irrigation and fertilization to obtain efficient and high-quality production.

## 4. Materials and Methods

### 4.1. Overview of the Test Area

**Figure 3:**
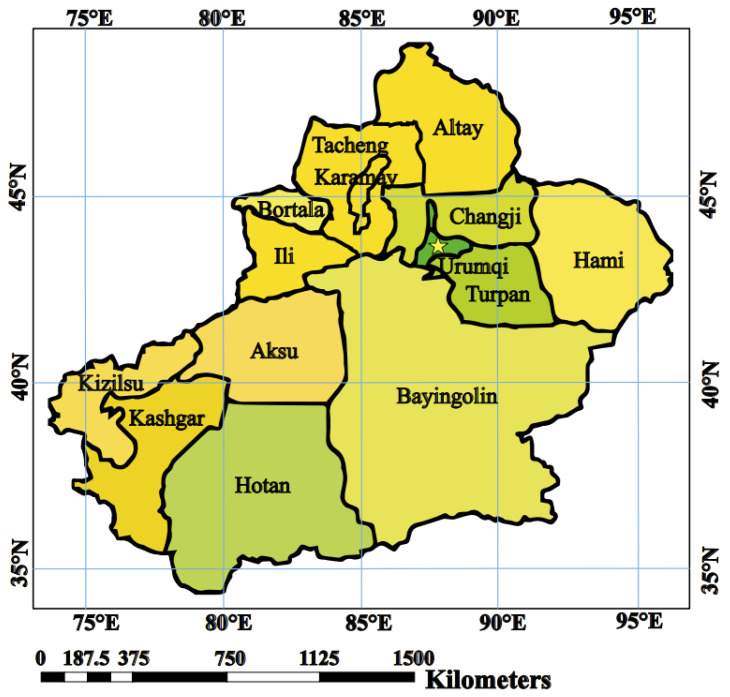
Schematic diagram of the test site.

**Figure 4:**
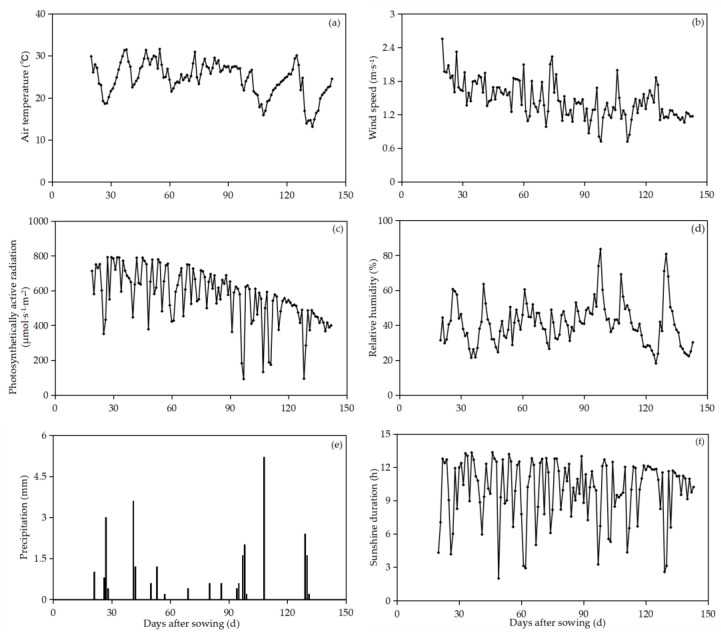
Daily variations in daily mean temperature (**a**), wind speed (**b**), photosynthetically active radiation (**c**), relative humidity (**d**), precipitation (**e**), and sunshine duration (**f**) during peanut growth period.

The experiment was carried out from May to October 2022 at Anningqu (87°30′ E, 43°58′ N, altitude of 590 m) (Figure 3). This area has a typical temperate, continental arid climate, and the climatic conditions are suitable for the growth and development of peanut. The average annual sunshine hours in the test area are 2700~2800 h, the effective accumulated temperature above 10 °C is 3000~3500 °C, the frost-free period is 170~179 d, the average annual rainfall is 200 mm, the average evaporation is 1750 mm, the groundwater depth is 7.5 m, and the soil texture is gray desert soil. The pH value of the topsoil in the test area is 7.8~8.0. The soil physical and chemical properties of the 0~60 cm soil layers are shown in Table 8. The daily variations in meteorological indicators during the growth period of peanut are shown in Figure 4.

### 4.2. Experimental Design

The test variety was Huayu 9610, and the fertility period was divided into five fertility stages according to the growth habit of peanut: the seedling stage, flowering–pegging stage, pod-setting stage, pod-filling stage, and harvesting stage [1].

**Figure 5:**
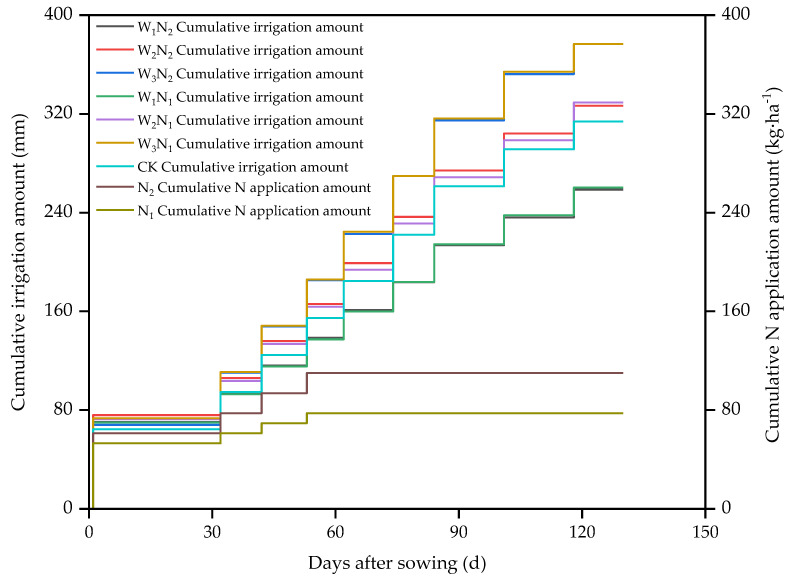
The actual irrigation and fertilization times, irrigation volumes, and nitrogen application rates during the peanut growth period. Note: W_1_, W_2_, and W_3_ represent irrigation water quotas of 22.5, 30, and 37.5 mm, respectively. N_1_ and N_2_ represent nitrogen application rates of 77.5 and 110 kg·ha^−1^, respectively. CK represents a 30 mm irrigation water quota and no nitrogen application.

Seven treatments were designed for the experiment and included two factors (irrigation water quota and nitrogen application rate) (Table 9). The three irrigation levels were W_1_ (irrigation water quota of 22.5 mm), W_2_ (irrigation water quota of 30 mm), and W_3_ (irrigation water quota of 37.5 mm). The two nitrogen application levels were N_1_ (nitrogen application rate of 77.5 kg·ha^−1^) and N_2_ (nitrogen application rate of 110 kg·ha^−1^). A control treatment (CK, irrigation water quota of 30 mm) did not include the application of nitrogen. The experiment was repeated five times for each treatment. An isolation ridge was built between every two plots to prevent water and fertilizer interactions between different treatments. The actual dates of irrigation and fertilization and the amounts of irrigation and nitrogen application are shown in Figure 5.

**Figure 6:**
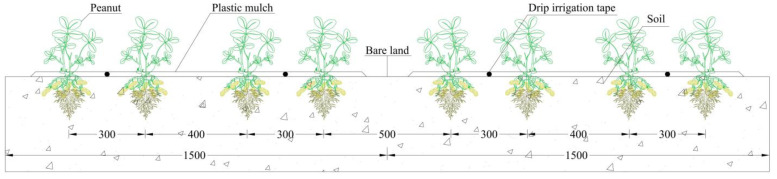
Layout of drip irrigation in the peanut field under film mulching (mm).

**Figure 7:**
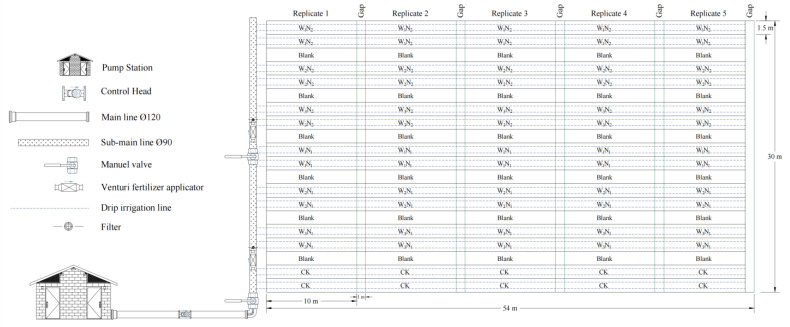
Layout of the field experiment. Note: W_1_, W_2_, and W_3_ represent irrigation water quotas of 22.5, 30, and 37.5 mm, respectively. N_1_ and N_2_ represent nitrogen application rates of 77.5 and 110 kg·ha^−1^, respectively. CK represents a 30 mm irrigation water quota and no nitrogen application. Blank stands for blank film.

The peanut planting pattern with drip irrigation under film mulching was 1 film with 2 belts and 4 rows (Figure 6); the average hole distance was 15 cm, and the planting density was 166,000 holes·ha^−1^. Sowing occurred on May 7 (dry sowing and wet emergence), the emergence of whole seedlings started on May 14, and the harvest occurred on September 27. Before sowing, the base compound fertilizer (N-P_2_O_5_-K_2_O = 15-15-15) was 300 kg·ha^−1^, and a nitrogen fertilizer (CO(NH_2_)_2_, with nitrogen content ≥ 46%) was applied with water during the growth period. Chemical control, spraying, and other agronomic measures were applied under a high-yield farmland management mode. A drip irrigation system under the film was used for irrigation, and the capillary was a labyrinth drip irrigation belt. The dripper flow rate was 3.2 L·h^−1^, and the dripper spacing was 30 cm. The test area was about 1620 m^2^ and was controlled by a branch pipe. The five plots of each treatment were arranged as a branch pipe unit (Figure 7). Gate valves and water meters were installed at the entrance of the unit. The irrigation water source was groundwater, and a water meter was used to control the irrigation amount for each treatment.

### 4.3. Observation Items and Methods

#### 4.3.1. Soil Moisture Content

The soil gravimetric method was used to determine stratification (0~20, 20~40, 40~60, 60~80, and 80~100 cm) before sowing, before irrigation, and after harvest. Considering the characteristics of wide- and narrow-row planting and the infiltration characteristics of the drip irrigation line source, four sampling points were selected for each treatment after irrigation and were directly below the center of 0 (film), 20 (inner row), 35 (under drip irrigation belt), and 70 (between film) cm. The method of Shen [42] was used to calculate the average soil moisture content of the profile to represent the average soil moisture content of the peanut field.

#### 4.3.2. Calculation of Field Yield and Water Consumption

After peanut harvest, three representative 6.67 m^2^ quadrats were selected for each treatment; the pods of these quadrats were stored in mesh bags and naturally dried and weighed, and their weight was converted into yield per hectare.

The water consumption of the peanuts in the experimental plot was calculated based on Formula (1) [43]. In addition, the groundwater depth of the test area during the growth period of the peanuts was greater than 7.5 m, so the groundwater recharge in the growing season could be counted as 0. There was no effective precipitation during the whole growth period of the peanuts in the test area, so *P*_0_ = 0. The observation data of soil moisture in the experimental field [1] showed that the irrigation quota was less than 37.5 mm, and the irrigation water had little effect on the soil moisture content of the soil layer below 60 cm. It could be considered that there was no deep leakage in the drip irrigation peanut field, and *D* = 0. Therefore, the equation for calculating the water consumption of the peanuts can be simplified as Equation (2):

(1) $$\mathrm{ET}=P_{0}+K+M\;-\;D+(W_{0}-W_{t})$$

(2) $$\mathrm{ET}=M+(W_{0}-W_{t})$$

where *ET* is the water consumption of the peanuts (mm); *P*_0_ is adequate precipitation (mm); *K* is groundwater recharge (mm); *M* is irrigation water (mm); *D* is deep seepage (mm); and *W*_0_ and *W_t_* represent the soil water storage at the beginning and at the end of the period, respectively.

#### 4.3.3. Determination of Plant Nutrient Content

The nitrogen content of the peanuts was determined using the Kjeldahl method [44].

#### 4.3.4. Water-Use Efficiency

Water-use efficiency and related indicators were calculated as follows:

(3) $$\mathrm{WUE}=Y/{\mathrm{ET}}_{a}$$

(4) $$\mathrm{IWUE}=Y/{\mathrm{ET}}_{a}$$

where *WUE* and *IWUE* denote the water-use efficiency and irrigation water-use efficiency (kg·m^−3^), respectively; *Y* is the pod yield of peanuts (kg·ha^−1^); *ET_a_* is the actual water consumption of peanuts during the whole growth stage (m^3^·ha^−1^); and *I* is the total amount of irrigation during the whole growth stage of peanuts during under-membrane drip irrigation, i.e., the irrigation quota (m^3^·ha^−1^).

#### 4.3.5. Nitrogen-Use Efficiency

Nitrogen-use efficiency and related indicators were calculated as follows:

(5) NUE=Y/A

(6) UPE=A/N

(7) NHI=AG/A

(8) NPFP=Y/N

where *NUE* is the nitrogen-use efficiency (kg·kg^−1^); *UPE* is the nitrogen uptake efficiency (kg·kg^−1^); *NHI* is the nitrogen harvest index; *NPFP* is the nitrogen partial factor productivity (kg·kg^−1^); *A* is the plant nitrogen accumulation (kg·ha^−1^); *AG* is the plant pod nitrogen accumulation (kg·ha^−1^); and *N* is the total amount of nitrogen applied to peanuts during under-membrane drip irrigation throughout the growth stage (kg·ha^−1^).

#### 4.3.6. Determination of Peanut Kernel Quality

Uniform pods were selected from naturally air-dried pods from each treatment, and the protein and oil contents of the treated kernels were determined using a near-infrared analyzer (NIRS^TM^ DS2500 F, Hillerød, Denmark).

#### 4.3.7. Multiple Linear Regression (MLR)

MLR is a statistical method that attempts to model the relationship between two or more interpretive variables (independent) and a response variable (dependent) by fitting a linear equation into the observed data. The model for MLR is

(9) $$y_{i}=a_{0}+a_{1}x_{1}+a_{2}x_{2}+\cdots +a_{k}x_{i}+e_{i}$$

where *y_i_* is the dependent variable; *a*_0_ is a constant (intercept); *x*_*i*,*k*_ denotes the independent variables; *a_k_* is the vector of regression coefficients (slope); and *e_i_* denotes random measurement errors.

#### 4.3.8. Multi-Objective Decision and Evaluation Based on the EWM-TOPSIS Method

Compared to the subjective weight method, the entropy weight method (EWM) produces an indicator weight value that is more reliable and accurate. According to the EWM, the indicator weights were calculated using the following steps: The technique for order preference by similarity to an ideal solution (TOPSIS) was used to identify a solution from a feasible solution set by defining the positive ideal solution and the negative ideal solution of the problem and selecting the ideal solution that was the most positive and furthest from the negative ideal solution. The process for the analysis is described below.

An evaluation index matrix of yield, quality, and water- and nitrogen-use efficiencies under different water and fertilizer treatments was established as follows:

(10) $$X=\left[\begin{array}{ccccc} x_{11} & \cdots & x_{1j} & \cdots & x_{1m}\\ \vdots & & \vdots & & \vdots \\ x_{i1} & \cdots & x_{\mathrm{ij}} & \cdots & x_{\mathrm{im}}\\ \vdots & & \vdots & & \vdots \\ x_{i1} & \cdots & x_{\mathrm{ij}} & \cdots & x_{\mathrm{im}} \end{array}\right]$$

Here, *x_ij_* represents the *j*th evaluation index of the *i*th treatment of the original data.

The evaluation index was standardized to unify the types and dimensions of the various indexes. The formulae are shown below.

For positive indicators, the following formula was used:

(11) $$y_{\mathrm{ij}}=\frac{x_{\mathrm{ij}}-\min\left(x_{j}\right)}{\max\left(x_{j}\right)-\min\left(x_{j}\right)}$$

For negative indicators, the following formula was used:

(12) $$y_{\mathrm{ij}}=\frac{\max\left(x_{j}\right)-x_{\mathrm{ij}}}{\max\left(x_{j}\right)-\min\left(x_{j}\right)}$$

The proportion (*P_ij_*) of the *j*th index represented by the *i*th treatment was calculated as follows:

(13) $$P_{\mathrm{ij}}=\frac{y_{\mathrm{ij}}}{\;\sum_{i=1}^{m}\;y_{\mathrm{ij}}}$$

The entropy value *e_j_* of the *j*th index was calculated as follows:

(14) $$e_{j}=\;-\frac{1}{ln_{n}}\Sigma_{i=1}^{n}p_{\mathrm{ij}}\ln p_{\mathrm{ij}}$$

The difference coefficient *g_j_* of the *j*th index was calculated as follows:

(15) $$g_{j}=1-e_{j}$$

The weight *W_j_* of the *j*th index was calculated as follows:

(16) $$W_{j}=\frac{g_{j}}{\sum_{i=1}^{m}\;g_{j}}$$

The weighted canonical matrix was calculated as follows:

(17) $$Z_{\mathrm{ij}}=y_{\mathrm{ij}}\times W_{j}$$

The ideal solution (*Z_ij_*^+^) and the negative ideal solution (*Z_ij_*^−^) were determined to form the ideal solution vector *Z* and the negative ideal solution vector *Z*^+−^, respectively, as follows:

(18) $$Z_{j}^{+}=\max\left(Z_{1j},Z_{2j},\ldots ,Z_{\mathrm{nj}}\right)\;$$

(19) $$Z_{j}^{-}=\min\left(Z_{1j},Z_{2j},\ldots ,Z_{\mathrm{nj}}\right)\;$$

The Euclidean distances *D* and *D*^+−^ were determined between the seven treatments and the negative ideal solution as follows:

(20) $$D_{i}^{+}=\sqrt{\sum_{j=1}^{n}\;(z_{\mathrm{ij}}-z_{j}^{+}{)}^{2}}$$

(21) $$D_{i}^{-}=\sqrt{\sum_{j=1}^{n}\;(z_{\mathrm{ij}}-z_{j}^{-}{)}^{2}}$$

The comprehensive benefit evaluation index *C_i_* of each treatment was calculated; that is, the proximity between the evaluation object and the optimal scheme was calculated as follows:

(22) $$C_{i}=\frac{D_{i}^{-}}{\;D_{i}^{+}+D_{i}^{-}}$$

#### 4.3.9. Meteorological Indicators

Meteorological data were continuously monitored by a standard automatic weather station located in an open field about 250 m away from the experimental field. Meteorological variables were recorded every 24 h, including photosynthetically active radiation (PAR, μmol·s^−1^·m^−2^), relative air humidity (RH, %), air temperature (Ta, °C), wind speed (Ws, m·s^−1^), precipitation (P, mm), and sunshine (SUN, h).

#### 4.3.10. Statistical Analyses

Excel 2016 was used to sort out and analyze the experimental data and draw the chart. An analysis of variance (ANOVA) was conducted to evaluate the effects of different irrigation water quotas (irrigation water quotas of 22.5, 30, and 37.5 mm, respectively, for W_1_, W_2_, and W_3_) and nitrogen application rates (nitrogen application rates of 0, 77.5, and 110 kg·ha^−1^, respectively, for CK, N_1_, and N_2_) on the parameters under study. The significance of different irrigation and nitrogen treatment effects was determined using the F-test, and comparisons of means were carried out using the least significant difference (LSD) test at the 5% level of significance.

## 5. Conclusions

The following conclusions are drawn based on the findings of our experimental study:

(1) The flowering–pegging stage and pod-setting stage are the key stages of peanut water requirement.
(2) When water consumption during the whole growth period of peanut is about 402.57 mm, a high yield can be achieved.
(3) The results of the multivariate linear regression analysis showed that the contribution rates of water-related factors to total water consumption, water-use efficiency, nitrogen-use efficiency, and pod yield were 98.5%, 83.7%, 45.6%, and 92.7%, respectively. The contribution rates of nitrogen-related factors were 16.9%, 34.4%, 50.5%, and 22.6%, respectively.
(4) A TOPSIS multi-objective comprehensive evaluation model was established by combining 13 indicators, and the final weight of each index was substituted to calculate the closeness degree, which was the largest for the W_3_N_2_ treatment at 0.7424. Under this treatment, the comprehensive index evaluation of the peanuts was the best, followed by the W_3_N_1_ treatment, while the W_1_N_1_ treatment showed the worst at only 0.2112.
(5) With the synergistic regulation of water and nitrogen, an irrigation quota of 37.5 mm, a nitrogen application rate of 110 kg·ha^−1^, and an irrigation period of 10~15 d constituted the best combination of water and nitrogen for peanut production under mulched drip irrigation in Xinjiang.

## Figures and Tables

**Table 1:** Effects of water and nitrogen regulation on the proportion of soil water consumption of peanuts under mulched drip irrigation.

Treatment	Total Water Consumption (mm)	Source of Water Consumption			
		Irrigation Capacity (mm)	Proportion (%)	Soil Water Consumption (mm)	Proportion (%)
W_1_N_2_	321.33	258.61	80.48	62.72	19.52
W_2_N_2_	360.38	326.67	90.65	33.71	9.35
W_3_N_2_	402.57	376.70	93.57	25.87	6.43
W_1_N_1_	329.81	260.35	78.94	69.46	21.06
W_2_N_1_	366.91	331.23	90.28	35.68	9.72
W_3_N_1_	407.60	378.73	92.92	28.87	7.08
CK	372.51	313.89	84.26	58.62	15.74

Note: W_1_, W_2_, and W_3_ represent irrigation water quotas of 22.5, 30 and 37.5 mm, respectively. N_1_ and N_2_ represent nitrogen application rates of 77.5 and 110 kg·ha^−1^, respectively. CK represents a 30 mm irrigation water quota and no nitrogen application.

**Table 2:** Effects of different treatments on the water consumption of peanuts under mulched drip irrigation in Xinjiang.

Treatment	Seedling Stage			Flowering–Pegging Stage			Pod-Setting Stage			Pod-Filling Stage		
	Water Consumption (mm)	Water Consumption Intensity (mm·d^−1^)	Water Consumption Percentage (%)	Water Consumption (mm)	Water Consumption Intensity (mm·d^−1^)	Water Consumption Percentage (%)	Water Consumption (mm)	Water Consumption Intensity (mm·d^−1^)	Water Consumption Percentage (%)	Water Consumption (mm)	Water Consumption Intensity (mm·d^−1^)	Water Consumption Percentage (%)
W_1_N_2_	48.26	1.72	15.02	102.43	3.53	31.88	131.70	3.56	40.99	38.95	0.87	12.12
W_2_N_2_	51.66	1.85	14.33	114.98	3.96	31.91	151.61	4.10	42.07	42.13	0.94	11.69
W_3_N_2_	50.34	1.80	12.50	121.56	4.19	30.20	176.37	4.77	43.81	54.30	1.21	13.49
W_1_N_1_	47.09	1.68	14.28	104.86	3.62	31.79	137.81	3.72	41.78	40.05	0.89	12.14
W_2_N_1_	50.43	1.80	13.74	120.08	4.14	32.73	153.47	4.15	41.83	42.93	0.95	11.70
W_3_N_1_	48.44	1.73	11.88	126.70	4.37	31.08	178.16	4.82	43.71	54.30	1.21	13.32
CK	49.52	1.77	13.29	122.90	4.24	32.99	155.60	4.21	41.77	44.49	0.99	11.94

Note: W_1_, W_2_, and W_3_ represent irrigation water quotas of 22.5, 30, and 37.5 mm, respectively. N_1_ and N_2_ represent nitrogen application rates of 77.5 and 110 kg·ha^−1^, respectively. CK represents a 30 mm irrigation water quota and no nitrogen application.

**Table 3:** Effects of water and nitrogen regulation on the nitrogen uptake efficiency and nitrogen-use efficiency of peanuts under mulched drip irrigation.

Treatment	Pod Nitrogen Accumulation (kg·ha^−1^)	Plant Nitrogen Accumulation (kg·ha^−1^)	Nitrogen Uptake Efficiency (kg·kg^−1^)	Nitrogen Harvest Index	Nitrogen-Use Efficiency (kg·kg^−1^)	Nitrogen Partial Factor Productivity (kg·kg^−1^)
W_1_N_2_	389.78 ± 72.32 cd	113.82 ± 23.51 c	1.03 ± 0.21 c	3.54 ± 0.83 b	24.15 ± 2.49 c	24.99 ± 2.58 d
W_2_N_2_	615.15 ± 69.85 b	241.85 ± 6.19 a	2.20 ± 0.06 a	2.54 ± 0.06 c	18.70 ± 0.36 c	41.12 ± 0.79 c
W_3_N_2_	734.89 ± 63.60 a	139.02 ± 1.47 b	1.26 ± 0.13 b	5.29 ± 0.06 a	38.10 ± 2.71 b	48.16 ± 3.43 b
W_1_N_1_	201.96 ± 4.87 e	57.02 ± 4.44 e	0.74 ± 0.06 d	3.56 ± 0.29 b	39.44 ± 0.17 b	29.02 ± 0.27 d
W_2_N_1_	440.63 ± 37.01 c	102.42 ± 8.44 cd	1.32 ± 0.11 b	4.32 ± 0.34 b	36.11 ± 4.16 b	47.72 ± 5.50 b
W_3_N_1_	396.96 ± 17.76 cd	93.83 ± 8.15 cd	1.21 ± 0.11 bc	4.25 ± 0.39 b	50.07 ± 1.66 a	60.61 ± 2.01 a
CK	330.81 ± 72.03 d	84.38 ± 11.17 d	-	3.97 ± 0.56 b	40.28 ± 6.67 b	-

Note: W_1_, W_2_, and W_3_ represent irrigation water quotas of 22.5, 30, and 37.5 mm, respectively. N_1_ and N_2_ represent nitrogen application rates of 77.5 and 110 kg·ha^−1^, respectively. CK represents a 30 mm irrigation water quota and no nitrogen application. Different lowercase letters indicate that the mean values are significantly different from one another at *p* ≤ 0.05.

**Table 4:** Effects of water and nitrogen on water consumption index of peanuts under mulched drip irrigation.

Index	Item	Intercept	Irrigation Water	Nitrogen Fertilizer	Coefficient of Determination	*F*
Water consumption	Regression coefficient	164.187	0.664	−0.142	0.976 **	122.74
	Standard error	14.302	0.043	0.053	-	-
	Partial correlation coefficient	-	0.985	−0.169	-	-
*WUE*	Regression coefficient	−0.397	0.004	0.002	0.767 *	10.882
	Standard error	0.310	0.001	0.001	-	-
	Partial correlation coefficient	-	0.837	0.344	-	-
*NUE*	Regression coefficient	14.533	0.099	−0.137	0.442	1.582
	Standard error	27.171	0.081	0.101	-	-
	Partial correlation coefficient	-	0.456	−0.505	-	-
Pod yield	Regression coefficient	−3436.64	20.96	6.381	0.895 **	26.575
	Standard error	1002.239	2.993	3.735	-	-
	Partial correlation coefficient	-	0.927	0.226	-	-

Note: “*” means significant (*p* ≤ 0.05); “**” means extremely significant (*p* ≤ 0.01).

**Table 5:** Effects of water and nitrogen regulation on peanut quality under mulched drip irrigation.

Treatment	Protein (%)	Oil Content (%)
W_1_N_2_	27.12 ± 0.12 b	40.18 ± 0.10 b
W_2_N_2_	27.37 ± 0.33 b	43.78 ± 1.20 a
W_3_N_2_	24.88 ± 0.88 c	45.42 ± 0.93 a
W_1_N_1_	26.65 ± 0.29 b	36.29 ± 0.71 c
W_2_N_1_	28.40 ± 0.73 a	38.30 ± 0.47 b
W_3_N_1_	25.12 ± 0.20 c	45.74 ± 2.24 a
CK	26.85 ± 0.58 b	43.86 ± 0.40 a

Note: W_1_, W_2_, and W_3_ represent irrigation water quotas of 22.5, 30, and 37.5 mm, respectively. N_1_ and N_2_ represent nitrogen application rates of 77.5 and 110 kg·ha^−1^, respectively. CK represents a 30 mm irrigation water quota and no nitrogen application. Different lowercase letters indicate that the mean values are significantly different from one another at *p* ≤ 0.05.

**Table 6:** Entropy values and weights calculated based on the entropy weight method.

**Index**	**x_1_**	**x_2_**	**x_3_**	**x_4_**	**x_5_**	**x_6_**	**x_7_**
Entropy	0.8722	0.8809	0.8748	0.8882	0.8571	0.8164	0.8725
Weight	0.0710	0.0662	0.0696	0.0622	0.0795	0.1021	0.0709
**Index**	**x_8_**	**x_9_**	**x_10_**	**x_11_**	**x_12_**	**x_13_**	
Entropy	0.8548	0.8387	0.8764	0.8548	0.8449	0.8695	
Weight	0.0807	0.0897	0.0687	0.0808	0.0862	0.0725	

**Table 7:** The comprehensive index evaluation results of peanuts under mulched drip irrigation based on TOPSIS.

**Treatment**	**x_1_**	**x_2_**	**x_3_**	**x_4_**	**x_5_**	**x_6_**	**x_7_**	**x_8_**	**x_9_**
W_1_N_2_	0.0195	0.0243	0.0121	0.0225	0.0130	0.0060	0.0161	0.0001	0.0112
W_2_N_2_	0.0642	0.0636	0.0001	0.0001	0.0593	0.0608	0.0503	0.0581	0.0458
W_3_N_2_	0.0710	0.0662	0.0431	0.0622	0.0795	0.1021	0.0709	0.0807	0.0897
W_1_N_1_	0.0001	0.0001	0.0460	0.0229	0.0001	0.0001	0.0001	0.0104	0.0001
W_2_N_1_	0.0365	0.0308	0.0386	0.0403	0.0378	0.0407	0.0440	0.0419	0.0347
W_3_N_1_	0.0527	0.0460	0.0696	0.0387	0.0638	0.0627	0.0595	0.0656	0.0650
CK	0.0258	0.0267	0.0479	0.0323	0.0300	0.0282	0.0271	0.0342	0.0285
**Treatment**	**x_10_**	**x_11_**	**x_12_**	**x_13_**	***D*+**	***D*−**	**Closeness Degree**	**Ranking**	
W_1_N_2_	0.0167	0.0145	0.0548	0.0298	0.2203	0.0812	0.2693	6	
W_2_N_2_	0.0375	0.0397	0.0610	0.0575	0.1313	0.1827	0.5818	3	
W_3_N_2_	0.0687	0.0808	0.0001	0.0701	0.0902	0.2600	0.7424	1	
W_1_N_1_	0.0001	0.0001	0.0434	0.0001	0.2542	0.0681	0.2112	7	
W_2_N_1_	0.0312	0.0311	0.0862	0.0154	0.1475	0.1516	0.5068	4	
W_3_N_1_	0.0417	0.0537	0.0058	0.0725	0.1095	0.2030	0.6497	2	
CK	0.0292	0.0278	0.0483	0.0581	0.1634	0.1283	0.4397	5	

Note: W_1_, W_2_, and W_3_ represent irrigation water quotas of 22.5, 30 and 37.5 mm, respectively. N_1_ and N_2_ represent nitrogen application rates of 77.5 and 110 kg·ha^−1^, respectively. CK represents a 30 mm irrigation water quota and no nitrogen application. *D*+ and *D*− represent the distance between the evaluation object and the positive ideal solution and the distance between the evaluation object and the negative ideal solution, respectively.

**Table 8:** Basic physical and chemical properties of test soil.

Soil Layer (cm)	Available P (mg·kg^−1^)	Available K (mg·kg^−1^)	Alkaline Hydrolysis N (mg·kg^−1^)	Organic Matter (g·kg^−1^)	Bulk Density (g·cm^−3^)
0–20	23.77	199.06	42.98	14.86	1.35
20–40	22.17	169.33	32.04	14.28	1.43
40–60	22.86	114.46	43.41	9.73	1.44

**Table 9:** Experimental design.

Treatment	Irrigation Quota (mm)					Irrigation Cycle (d)				N Fertilizer Application Rate (kg·ha^−1^)
	Sowing-Emergence Stage	Seedling Stage	Flowering–Pegging Stage	Pod-Setting Stage	Pod-Filling Stage	Seedling Stage	Flowering–Pegging Stage	Pod-Setting Stage	Pod-Filling Stage	
W_1_N_2_	45	-	22.5	22.5	22.5	-	10	10	15	110
W_2_N_2_	45	-	30	30	30	-	10	10	15	110
W_3_N_2_	45	-	37.5	37.5	37.5	-	10	10	15	110
W_1_N_1_	45	-	22.5	22.5	22.5	-	10	10	15	77.5
W_2_N_1_	45	-	30	30	30	-	10	10	15	77.5
W_3_N_1_	45	-	37.5	37.5	37.5	-	10	10	15	77.5
CK	45	-	30	30	30	-	10	10	15	0

## Data Availability

The data are contained within the article.
